# Right Heart and Wrong Rhythm: Atrial Flutter in Dextrocardia

**DOI:** 10.7759/cureus.42177

**Published:** 2023-07-20

**Authors:** Roy Lim, Navdeep Bais, Furkhan Ali, Reejeen Monsalve, Brian Denney

**Affiliations:** 1 Internal Medicine, Mount Sinai Hospital, Chicago, USA; 2 Medicine, Ross University School of Medicine, Miramar, USA; 3 Internal Medicine, Our Lady of Fatima University, Valenzeula, PHL; 4 General Medicine, Cebu Velez General Hospital, Cebu City, PHL

**Keywords:** atrial fibrillation, atrial tachycardia, arrhythmia, situs inversus, dextrocardia, atrial flutter

## Abstract

Atrial flutter is characterized by rapid atrial activity, causing an abnormal heart rhythm. Recognition and prompt management are of utmost importance since this cardiac arrhythmia could increase the risk of thromboembolic stroke and atrial fibrillation, which may lead to disability and death. Risk factors include myocardial infarction, surgery, medication, and structural heart abnormalities. One distinctive structural abnormality is dextrocardia. Herein, we present a case of a 47-year-old male who initially complains of difficulty in ambulation. Further workup showed atrial flutter with rapid ventricular response on electrocardiogram (ECG) and dextrocardia on imaging. This case tackles the possible association between dextrocardia and arrhythmias, which was an atrial flutter, its management, and treatment outcomes.

## Introduction

Atrial flutter, a supraventricular abnormal cardiac rhythm, is one of the most common arrhythmias characterized by an atrial rate of 300 beats per minute (bpm) and a variable or fixed ventricular rate [1]. Being the second most frequent arrhythmia next to atrial fibrillation, it is becoming more and more prevalent globally [2]. Many risk factors can cause atrial flutter, both modifiable and unmodifiable. These include race, age, congenital heart failure, rheumatic valve disease, prior cardiac surgery, congenital heart disease, and structural cardiac abnormalities [3]. It is a potentially high-risk complication for patients with structural heart abnormalities. One such abnormality is dextrocardia. Herein, we present a patient with dextrocardia and secondarily developed atrial flutter.

Dextrocardia is a rare congenital condition in which the heart is on the right side of the thoracic cavity instead of its normal position on the left side [4]. It is often seen concurrently with other congenital anomalies, including situs inversus, a condition in which the organs in the chest and abdomen are not in their usual anatomical positions; however, it is inverted and positioned on the opposite side of the body [4,5]. Dextrocardia is seen in one out of every 12,000 pregnancies [4]. Dextrocardia can be a harmless condition, and patients can live a normal life until this finding is discovered. Most people will continue to live a normal life after its discovery. However, since dextrocardia can pose a potential risk factor for arrhythmias, there is a chance that a person can develop atrial flutter [6].

Our patient, in this case, presented with persistent pain in the right lower extremity. In addition, the patient had a history of persistent tachycardia with no prior consults done nor any medications taken. Workup revealed an incidental finding of a congenital heart anomaly, which is dextrocardia in this case. Arising from this structural heart anomaly was an atrial flutter with the rapid ventricular response (RVR) making this case unusual. A treatment regimen was initiated in this patient, which resulted in a reversion to sinus rhythm, and a plan for cardioversion is in place. With no set treatment guidelines for atrial flutter in dextrocardia, this case examines the association of dextrocardia, atrial flutter, and a potential treatment method.

## Case presentation

This case involves a 47-year-old male with situs inversus with a history of hypertension, poor medical compliance, and a gunshot wound in the right lower extremity. He presented in the emergency department for trouble walking due to pain localized in his right calf (pain scale 7-8/10) that progressed for over a month. He reported that the pain was worse when walking and improved at rest. Of note, he revealed a history of persistent tachycardia for which he took no medication.

**Figure 1:**
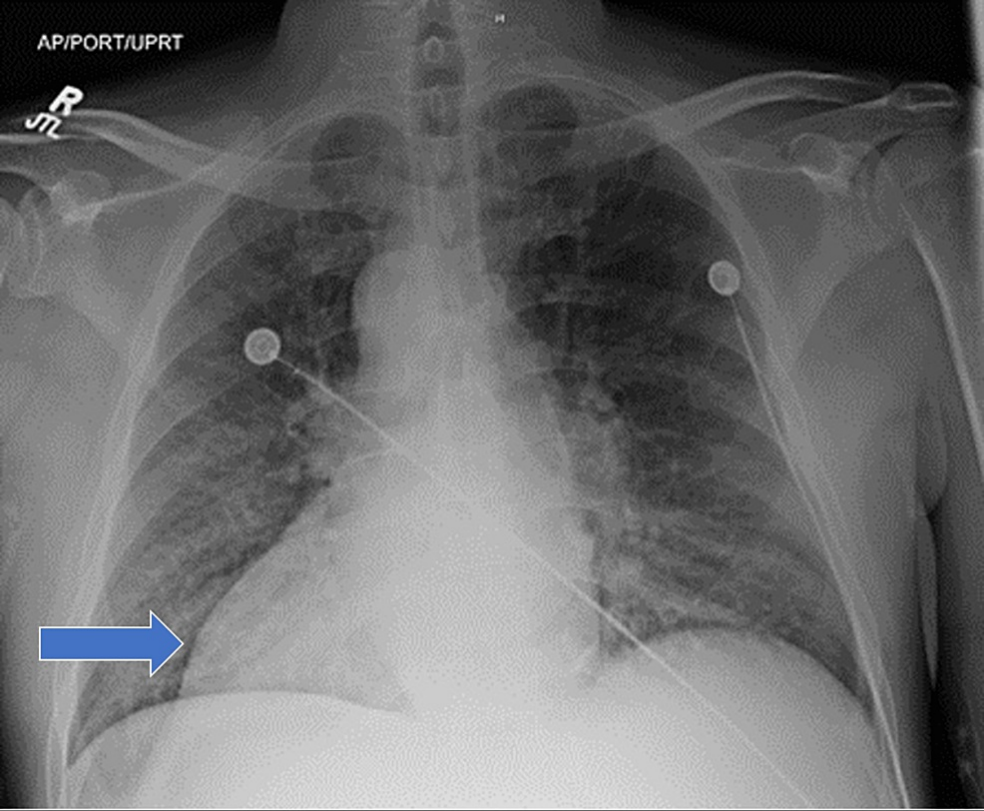
Chest x-ray AP view showed a cardiac malposition, dextroversion, in which the apex of the heart is pointing to the right (blue arrow)

**Figure 2:**
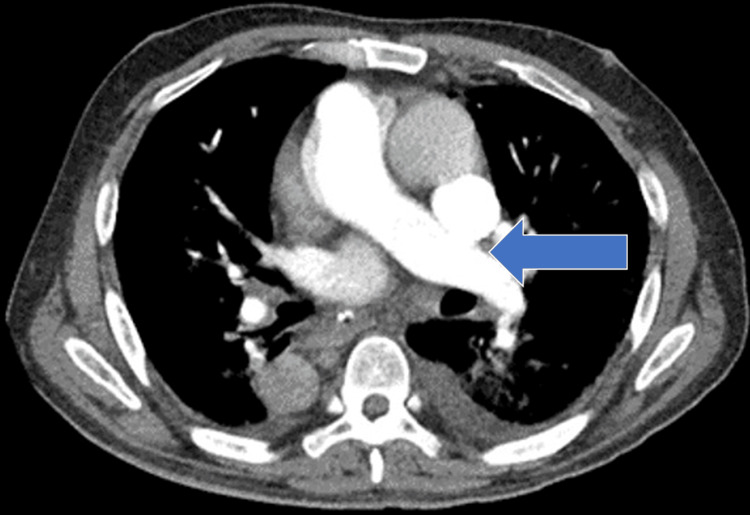
Chest computed tomography angiography (axial view) showed no filling defects which denotes absence of thrombus in the pulmonary vasculature (blue arrow)

On workup, the patient’s chest radiograph in Figure 1 showed dextrocardia with situs inversus, and a computed tomography scan of the chest in Figure 2 showed no evidence of pulmonary embolism.

**Figure 3:**
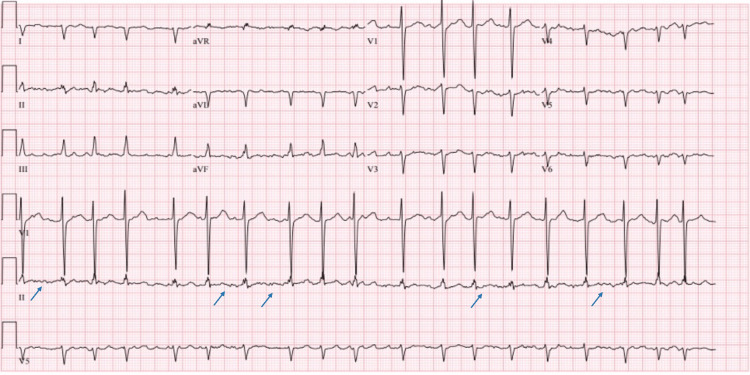
ECG showed a rapid atrial rate with irregular rhythm, with narrow QRS complex, and a characteristic saw-tooth appearance of the p-waves typical of atrial flutter (blue arrow)

A lower extremity venous duplex scan revealed minimal nonocclusive non-flow limiting thrombus at the distal right superficial femoral and popliteal veins. The patient was given a therapeutic dose of low molecular weight heparin (LMWH). Computed tomography pulmonary angiogram (CTPA) was done which was negative for pulmonary embolism but incidentally found an aneurysmal dilatation of the ascending aorta measuring 4.1cm. An electrocardiogram (ECG) in Figure 3 showed atrial flutter with rapid ventricular response.

The patient received an intravenous nondihydropyridine calcium channel blocker that successfully converted the atrial flutter to sinus rhythm. After the conversion, the patient was started on oral beta blockers. An echocardiogram was performed, and the results showed mild left ventricular hypertrophy, mild mitral valve regurgitation, and an ejection fraction of 60% to 65%. The patient left the hospital against medical advice. The cardiology team recommended regular follow-up appointments, including a future cardioversion procedure, which is yet to be scheduled.

## Discussion

In this case report, our patient presented with a structural heart abnormality causing cardiac arrhythmia. The patient had persistent tachycardia, which on ECG revealed findings characteristic of atrial flutter with an RVR. On further workup, a chest x-ray revealed a cardiac apex pointing to the right, typical of dextrocardia. Dextrocardia refers to the malpositioning of the heart in the right thoracic cavity [6]. Its exact etiology needs to be better understood; however, it is thought to result from anomalies occurring in the embryonic development of the heart [4]. This condition is commonly associated with situs inversus, when all the internal organs in the thorax and abdomen are in the mirror position of their normal anatomical sides [6]. In most cases, this condition is an incidental finding [4]. The malpositioning of the heart may predispose patients to certain arrhythmias, such as atrial flutter and atrial fibrillation, which can cause later complications. In a retrospective study by Naaraayan et al. in 2016 using their National Inpatient Sample (NIS) Database, the investigators concluded that patients with dextrocardia were more likely to have arrhythmias, especially atrial fibrillation atrial flutter, than those patients without [6].

Many risk factors, modifiable or nonmodifiable, predispose the patient to atrial flutter. One risk factor of our patient is hypertension, which was poorly controlled, likely due to non-compliance with medications. In imaging studies, a cardiac structural anomaly was also evident, dextrocardia, which predisposes the patient to cardiac arrhythmias. Race and age posed to be potential nonmodifiable risk factors. Modifiable risk factors include hypertension, diabetes, smoking, alcohol, obesity, physical activity, psychological stress, and potentially social factors [7]. Along with social factors, chronic health conditions can be risk factors for atrial flutter. Some examples are prior cardiac infections, heart failure, and myocardial infarction. Physicians managing the patients should be able to identify underlying risk factors for early diagnosis and provide individualized care.

Since atrial flutter can lead to atrial fibrillation, preventive measures, and prompt medical treatment is necessary as soon as diagnosed. The four main goals in treating atrial flutter include rate control, rhythm control, maintenance, and post-treatment prevention of any potential emboli [7,8]. In this case, the patient received a combination rate and rhythm control regimen, specifically, IV non-dihydropyridine calcium channel blocker and oral beta blocker. Hence, this patient was managed like a patient with no anatomical anomaly.

Conversion and maintenance of the sinus rhythm are vital [8], which may be achieved with electrical cardioversion for a hemodynamically unstable patient [9], pharmacological cardioversion with antiarrhythmic drugs (e.g., amiodarone, class IA and IC, calcium channel blockers, and beta-blockers) for hemodynamically stable patients [10], and ultimately catheter ablation for those patients who have contraindications to these drugs [11]. According to Shah et al., managing atrial flutter with rate control includes using either a calcium channel blocker like verapamil or a beta-blocker like metoprolol. On the other hand, Class III potassium channel blocker antiarrhythmics dofetilide and ibutilide can be used for rhythm control. Cardioversion is an option if rhythm or rate control cannot be achieved [12]. Anticoagulation is also necessary due to increased thromboembolic events in patients with atrial flutter [8]. There is a similar risk for stroke in patients with atrial flutter to those with atrial fibrillation [13]. A scoring system such as CHADS2-VASc helps to stratify their risk of developing embolic strokes [14].

There are currently no treatment guidelines for managing atrial flutter in dextrocardia. Management is optimized on a case-by-case basis. Carter et al. reported using radiofrequency ablation to terminate atrial flutter in a patient with dextrocardia [15]. Atrial fibrillation and atrial flutter can occur concurrently, as in the case of a 50-year-old male reported by Ulus et al., which presented as dextrocardia with associated situs inversus [16]. Ablation for both atrial fibrillation and atrial flutter successfully converted such arrhythmias to a sinus rhythm [16]. Pott et al. presented the benefit of a biventricular pacemaker placement in a patient with dextrocardia and persistent left superior vena cava [17]. Of note, another case of dextrocardia that progressed to ventricular tachycardia and ventricular fibrillation was managed with an implantable cardioverter-defibrillator [18]. As mentioned above, the studies demonstrated a wide spectrum of treatments for dextrocardia with an associated arrhythmia. However, specific guidelines can be developed to guide clinicians in practice.

## Conclusions

Since dextrocardia is a type of structural heart disease, there is a higher risk of arrhythmias such as atrial flutter and potentially atrial fibrillation. Dextrocardia is often associated with other anomalies, such as situs inversus. Our case report is one of the few that support the possible link between dextrocardia with situs inversus and arrhythmias such as atrial flutter. Thus, it is important to monitor patients diagnosed with dextrocardia and situs inversus for the development of arrhythmias. Considering this patient initially presented with tachycardia, emphasis on patient education about their increased risk and signs and symptoms that warrant a physician consultation is paramount.

There is a gamut of treatment modalities for dextrocardia with an associated cardiac arrhythmia. Due to the rarity of the case, we recommend that further analysis of the association between dextrocardia and situs inversus be done to provide possible preventive avenues and better therapeutic outcomes. In addition, regular follow-up and adherence to treatment plans are important to minimize the risk of complications.
